# Tuberoplasty reduces resistance force in dynamic shoulder abduction for irreparable rotator cuff tears: a cadaveric biomechanical study

**DOI:** 10.1186/s13018-024-04740-w

**Published:** 2024-06-08

**Authors:** Zhiyao Li, Lifeng Ma, Yike Dai, Heyong Yin, Naicheng Diao, Jingxin Zhang, Jizhou Zeng, Ai Guo

**Affiliations:** 1 Department of Orthopedics, Beijing Friendship Hospital, Capital Medical University, Yong’an road, Xicheng District, Beijing, 101149 China; 2 Department of Orthopedics, Beijing Luhe Hospital, Capital Medical University, No. 82 Xinhua South Road, Tongzhou District, Beijing, 101149 China; 3 Beijing Key Laboratory of Fundamental Research on Biomechanics in Clinical Application, Beijing, 100069 China

**Keywords:** Irreparable cuff tears, Tuberoplasty, Resistance force, Biomechanics

## Abstract

**Background:**

Arthroscopic tuberoplasty is an optional technique for managing irreparable rotator cuff tears. However, there is a lack of studies investigating the resistance force during shoulder abduction in cases of irreparable rotator cuff tears and tuberoplasty.

**Hypotheses:**

In shoulders with irreparable rotator cuff tears, impingement between the greater tuberosity (GT) and acromion increases the resistance force during dynamic shoulder abduction. Tuberoplasty is hypothesized to reduce this resistance force by mitigating impingement.

**Study design:**

Controlled laboratory study.

**Methods:**

Eight cadaveric shoulders, with a mean age of 67.75 years (range, 63–72 years), were utilized. The testing sequence included intact rotator cuff condition, irreparable rotator cuff tears (IRCTs), burnishing tuberoplasty, and prosthesis tuberoplasty. Burnishing tuberoplasty refers to the process wherein osteophytes on the GT are removed using a bur, and the GT is subsequently trimmed to create a rounded surface that maintains continuity with the humeral head. Deltoid forces and actuator distances were recorded. The relationship between deltoid forces and actuator distance was graphically represented in an ascending curve. Data were collected at five points within each motion cycle, corresponding to actuator distances of 20 mm, 30 mm, 40 mm, 50 mm, and 60 mm.

**Results:**

In the intact rotator cuff condition, resistance forces at the five points were 34.25 ± 7.73 N, 53.75 ± 7.44 N, 82.50 ± 14.88 N, 136.25 ± 30.21 N, and 203.75 ± 30.68 N. In the IRCT testing cycle, resistance forces were 46.13 ± 7.72 N, 63.75 ± 10.61 N, 101.25 ± 9.91 N, 152.5 ± 21.21 N, and 231.25 ± 40.16 N. Burnishing tuberoplasty resulted in resistance forces of 32.25 ± 3.54 N, 51.25 ± 3.54 N, 75.00 ± 10.69 N, 115.00 ± 10.69 N, and 183.75 ± 25.04 N. Prosthesis tuberoplasty showed resistance forces of 29.88 ± 1.55 N, 49.88 ± 1.36 N, 73.75 ± 7.44 N, 112.50 ± 7.07 N, and 182.50 ± 19.09 N. Both forms of tuberoplasty significantly reduced resistance force compared to IRCTs. Prosthesis tuberoplasty further decreased resistance force due to a smooth surface, although the difference was not significant compared to burnishing tuberoplasty.

**Conclusion:**

Tuberoplasty effectively reduces resistance force during dynamic shoulder abduction in irreparable rotator cuff tears. Prosthesis tuberoplasty does not offer a significant advantage over burnishing tuberoplasty in reducing resistance force.

**Clinical Relevance:**

Tuberoplasty has the potential to decrease impingement, subsequently reducing resistance force during dynamic shoulder abduction, which may be beneficial in addressing conditions like pseudoparalysis.

## Introduction

The rotator cuff plays a crucial role in collaborating with the deltoid muscle to maintain balance in force couples around the glenohumeral joint. These cuff muscles contribute to stabilizing the joint fulcrum, allowing the deltoid to efficiently elevate and abduct the shoulder [1, 2]. Tendon tears within the cuff can adversely affect the ability to maintain the native position of the humeral head. In cases of irreparable rotator cuff tears (IRCTs), the absence of cuff tendon integrity leads to superior migration of the humeral head and impingement between the greater tuberosity (GT) and the acromion [3]. Addressing subacromial impingement is a primary objective in the management of IRCTs. Various surgical techniques, such as subacromial balloon, superior capsular reconstruction (SCR), and others [4–6], aim to eliminate subacromial impingement by preserving a normal fulcrum.

Arthroscopic burnishing tuberoplasty emerges as a treatment option for IRCTs, with the goal of reducing subacromial impingement through acromiohumeral articulation [7–10]. Lee BG et al. Descrided their technique of arthroscopic tuberoplasty [8]. Under the arthroscope, the soft tissue around the greater tuberosity was removed with a shaver, and the bone protruding from the greater tuberosity was trimmed with a high-speed bur. At this step, no matter how the arm is positioned, the bone should be trimmed in a round shape to prevent collision between the acromion and the greater tuberosity. From the anterior aspect, the cortical bone of the lateral tubercle in the bicipital groove was removed sufficiently, with the remaining bone being shaped round. However, there is a lack of biomechanical studies on this technique. The primary objective of this study is to assess the resistance force during dynamic shoulder abduction in irreparable rotator cuff tears, both before and after burnishing tuberoplasty. To further alleviate subacromial impingement, we investigated a procedure known as prosthesis tuberoplasty. Building on our previous radiological study, which provided precise measurements of GT size [11], the secondary objective is to evaluate the resistance force during dynamic shoulder abduction following prosthesis tuberoplasty. Our hypothesis posits that burnishing tuberoplasty will reduce resistance forces compared to IRCTs, and prosthesis tuberoplasty will enhance this efficacy.

## Methods

Specimen: A total of eight paired cadaveric shoulders, obtained from two men and two women, were utilized in this study. The shoulders were severed at the medial border of the scapula and had a mean age of 67.75 years (range, 63–72 years). The cadavers were preserved using a saturated salt solution [12], maintaining a texture and tissue consistency similar to that of fresh-frozen specimens. Gross examination during dissection confirmed intact rotator cuffs, and cartilage was inspected through a small incision in the rotator interval. All specimens were free of glenohumeral osteoarthritis. The muscles of the supraspinatus, infraspinatus, teres minor, subscapularis, and deltoid were preserved, while other soft tissues were removed, preserving the glenohumeral joint capsule, coracoacromial, and coracohumeral ligaments [2].

**Fig. 1:**
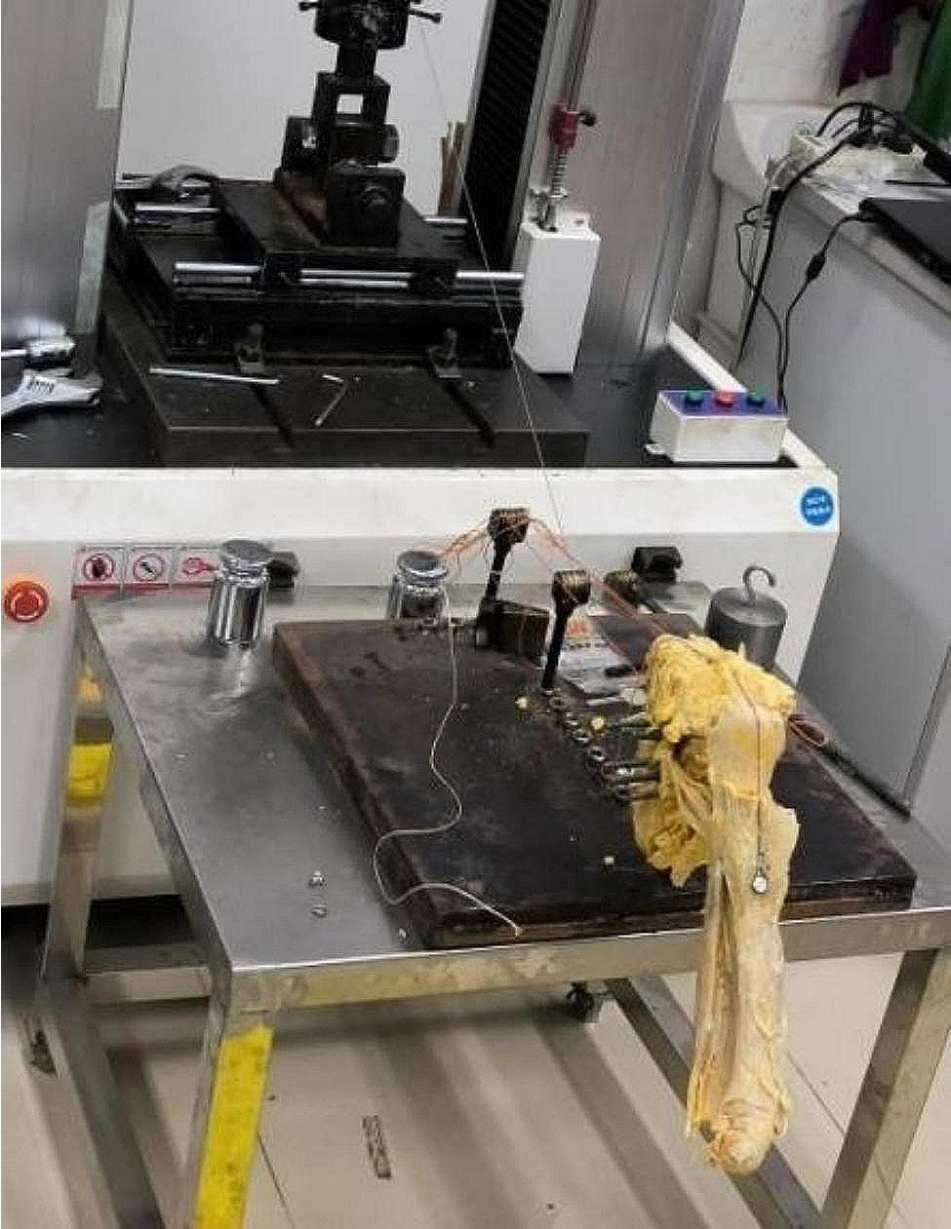
The customed equipment consisted of a baseplate and several pulleys for changing the direction of the steel wire, the steel wire connects the deltoid tubercle and the actuator

Simulator: The shoulder simulator comprised custom equipment for scapula fixation and an actuator (WDW4100, Changchun Kexin Testing Instruments Co. LTD, China). The custom equipment included a base plate made of steel with screw fixation holes and pulleys for altering the direction of the steel wire. The scapula was secured onto the base plate with a 15-degree glenoid inclination. A 2-mm steel wire was linked to the deltoid tuberosity via a screw, passing through the anterolateral corner of the acromion in parallel to the direction of the supraspinatus (SSP). The wire traversed three fixed pulleys before being fixed to the actuator. The actuator allowed for uniform motion at speeds ranging from 0 to 200 mm per minute, simultaneously measuring the resistance force. The actuator position was controllable and recorded in real-time throughout the loading cycle, with load cells recording the forces. The software generated a loading curve, where the horizontal axis represented the distance moved by the actuator. This distance had a positive correlation with the shoulder’s abduction angle, with every 10 mm corresponding to approximately 15 degrees of abduction. The vertical axis of the curve represented the resistance force, correlating with the contractile force of the deltoid muscle. Thus, the curve depicted the relationship between resistance force and the degree of shoulder abduction. Deltoid insertion at the deltoid tuberosity were dissected, and a metal screw served as the anchor for the steel wire from the actuator (Fig. 1).

### Testing cycle

This study comprised four testing cycles: intact rotator cuff condition, irreparable rotator cuff tears (IRCTs) condition, burnishing tuberoplasty, and prosthesis tuberoplasty.

In the intact rotator cuff condition, cuff insertions were identified and marked with No. 2 FiberTape (Arthrex, Naples, FL) to facilitate muscle loading. Muscle loading was achieved by applying weights using pulleys, with the weight adjusted to match previous biomechanical studies, considering the cross-sectional area of each muscle. Specifically, 10 N for the subscapularis, 5 N for the infraspinatus, 5 N for the teres minor, and 5 N for the supraspinatus (SSP) were applied to establish a balanced shoulder state [13, 14].

**Fig. 2:**
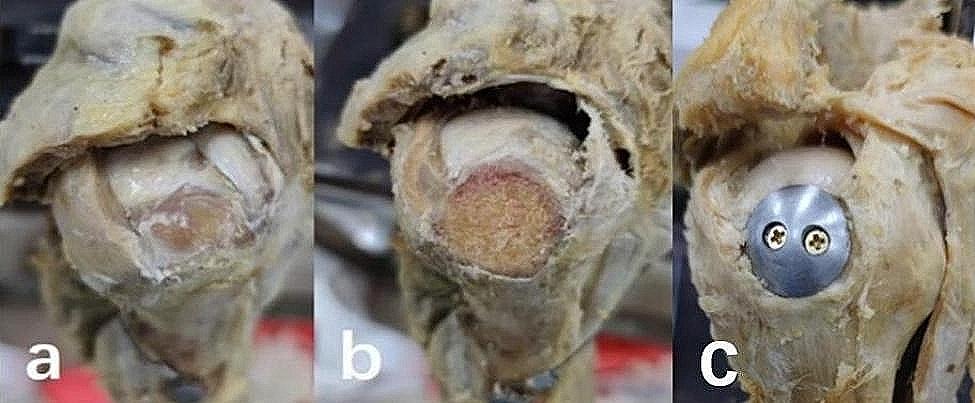
The specimen of IRCTs, burnishing tuberoplasty and prosthesis tuberoplasty

For the IRCTs condition, all parts of the SSP and superior capsule were removed. The superior half of the infraspinatus (ISP) was also excised. The entire insertion of SSP and the superior half of ISP on the greater tuberosity (GT) were cleared, exposing the cortex of GT. The static loading on the subscapularis, infraspinatus, and teres minor remained unchanged (Fig. 2a).

In the burnishing tuberoplasty condition, the GT was burnished to form an arc shape, allowing the humeral head to smoothly extend to the lateral cortex. The static loading on the subscapularis, infraspinatus, and teres minor remained unchanged (Fig. 2b).

In the prosthesis tuberoplasty condition, a prosthesis was designed based on a previous study [11]. The prosthesis, cut from a stainless-steel ball with an arc structure (3 cm in diameter), featured a central angle of 90° and two holes for screw fixation. The GT was burnished to create a flat surface, and the prosthesis was secured with two screws (Fig. 2c).

Testing commenced with the intact rotator cuff condition, followed by the IRCTs condition, burnishing tuberoplasty, and prosthesis tuberoplasty. Prior to testing, each specimen underwent passive full range of motion exercises 20 times to tension soft tissues, including ligaments, tendons, and capsules. Maximum abduction was achieved in the intact specimen. The actuator’s native position was established at 0 degrees of abduction. During testing, the actuator speed was set at 150 mm/min, and both the moving distance of the actuator and resistance forces were recorded. “Maximal” abduction was defined when the actuator moved more than 60 mm, corresponding to the humerus achieving an abduction angle of approximately 90 degrees. After each motion cycle, the specimen was returned to the starting position, and the distance of the moving actuator and resistance force were recorded throughout the four testing cycles.

### Statistical analysis

Deltoid forces and actuator distance were documented, and their relationship was depicted in an ascending curve. Data were selected at five points for each motion cycle, corresponding to actuator distances of 20 mm, 30 mm, 40 mm, 50 mm, and 60 mm. Results are presented as means with 95% confidence intervals (CIs). The adjusted alpha level for statistical significance was set at 0.05. Statistical analyses were conducted using SPSS version 19.0 (IBM Corp, Armonk, NY, USA), employing ANOVA with post-hoc tests to compare differences between groups. A *p*-value less than 0.05 was considered significant.

## Results

**Fig. 3:**
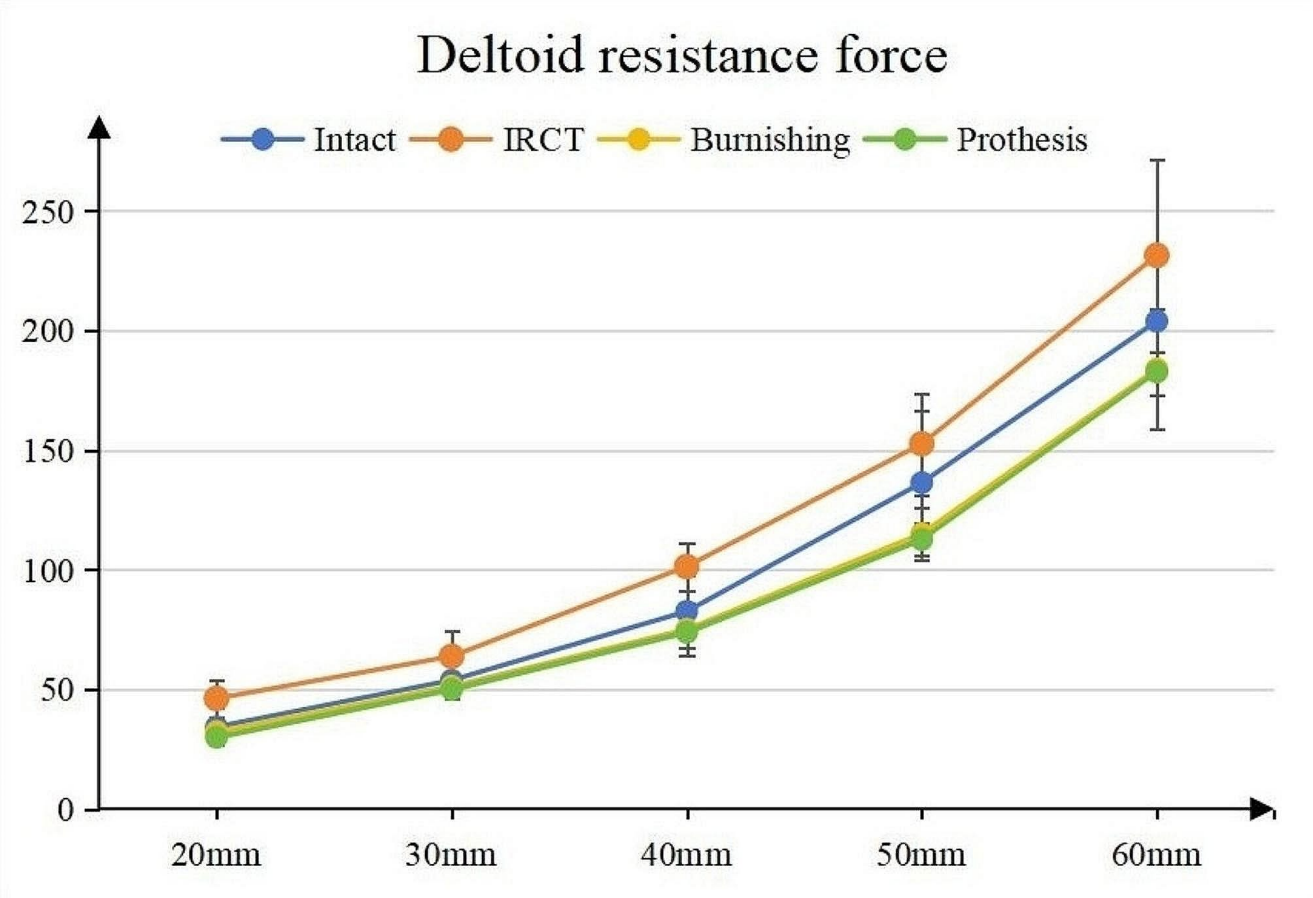
The deltoid loading curve during the shoulder abduction

**Fig. 4:**
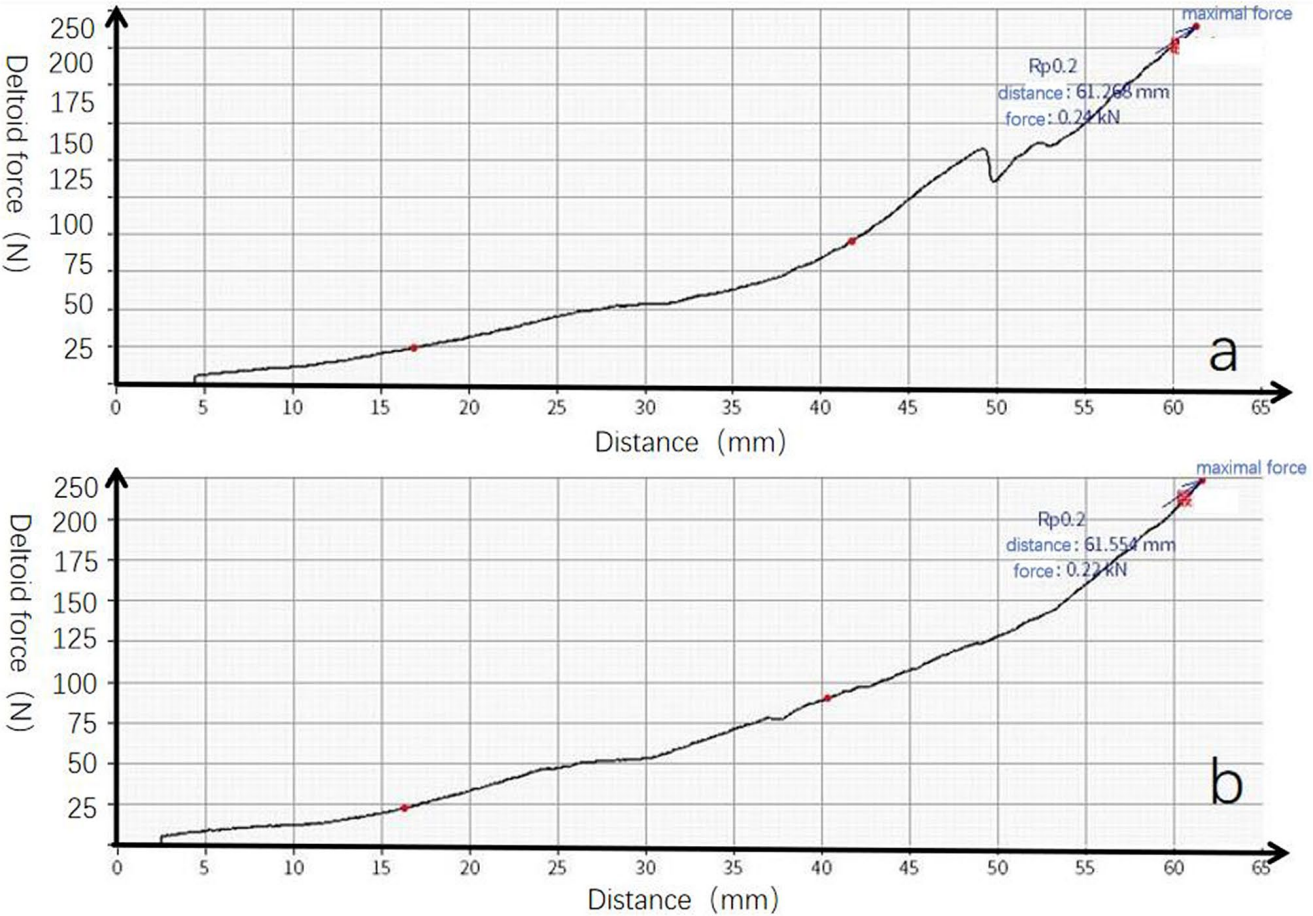
Loading curve of deltoid resistance force. (**a**) There was an obvious “bumpier” during the curve of SSP removal. (**b**) The “bumpier” disappeared, and the curve became smooth after tuberoplasty

In every testing cycle, the resistance force gradually increased as the actuator moved, as depicted in Fig. 3. During the IRCTs testing cycle, the loading curve exhibited noticeable irregularities, appearing “bumpier” (Fig. 4a). Upon polishing or replacing the GT, the irregularities disappeared, resulting in a smoother curve (Fig. 4b).

The average resistance forces and actuator distances are presented in Table 1. The initial testing cycle involved intact supraspinatus (SSP) with a load of 30 N. In the normal condition testing cycle, the resistance forces at five points were 34.25 ± 7.73 N, 53.75 ± 7.44 N, 82.50 ± 14.88 N, 136.25 ± 30.21 N, and 203.75 ± 30.68 N. In the testing cycle of irreparable rotator cuff tears (IRCTs), the resistance forces at the same points were 46.13 ± 7.72 N, 63.75 ± 10.61 N, 101.25 ± 9.91 N, 152.5 ± 21.21 N, and 231.25 ± 40.16 N. The resistance forces substantially increased, reaching the highest values among the four conditions. Resistance forces gradually decreased in subsequent testing cycles of burnishing tuberoplasty and prosthesis tuberoplasty. In the burnishing tuberoplasty testing cycle, the resistance forces at the five points were 32.25 ± 3.54 N, 51.25 ± 3.54 N, 75.00 ± 10.69 N, 115.00 ± 10.69 N, and 183.75 ± 25.04 N. In the prosthesis tuberoplasty testing cycle, the resistance forces at the five points were 29.88 ± 1.55 N, 49.88 ± 1.36 N, 73.75 ± 7.44 N, 112.50 ± 7.07 N, and 182.50 ± 19.09 N. The resistance force was the lowest in the prosthesis tuberoplasty condition.

**Table 1:** The resistance force in different condition of rotator cuff during the actuator moved (N)

	20 mm	30 mm	40 mm	50 mm	60 mm
Intact RC with loading 30 N(*n* = 8)	34.25 ± 7.73	53.75 ± 7.44	82.50 ± 14.88	136.25 ± 30.21	203.75 ± 30.68
IRCTs(*n* = 8)	46.13 ± 7.72	63.75 ± 10.61	101.25 ± 9.91	152.5 ± 21.21	231.25 ± 40.16
Burnising tuberoplasty(*n* = 8)	32.25 ± 3.54	51.25 ± 3.54	75.00 ± 10.69	115.00 ± 10.69	183.75 ± 25.04
Prosthesis tuberoplasty(*n* = 8)	29.88 ± 1.55	49.88 ± 1.36	73.75 ± 7.44	112.50 ± 7.07	182.50 ± 19.09

The comparison of resistance forces between burnishing arthroplasty and the other testing cycles is presented in Table 2. When compared to the intact rotator cuff cycle, the differences were not significant. However, when compared to the cycle of irreparable rotator cuff tears (IRCTs), the differences were significant at every point. Conversely, when compared to the cycle of prosthesis tuberoplasty, the differences were not significant.

**Table 2:** Comparison of resistance force between burnishing arthroplasty and other testing cycles

	20 mm	30 mm	40 mm	50 mm	60 mm
Intact	F = 1.113, *p* = 0.309	F = 0.737, *p* = 0.405	F = 1.340, *p* = 0.266	F = 3.518, *p* = 0.082	F = 2.041, *p* = 0.175
IRCTs	F = 24.567, *p* = 0.000	F = 10.000, *p* = 0.007	F = 25.941, *p* = 0.000	F = 19.937, *p* = 0.001	F = 8.061, *p* = 0.013
Prothesis tuberoplasty	F = 1.014, *p* = 0.331	F=-1.005, *p* = 0.322	F = 0.074, *p* = 0.790	F = 0.304, *p* = 0.590	F = 0.013, *p* = 0.912

## Discussion

We hypothesized that tuberoplasty would prevent impingement between the GT and the acromion in cases of irreparable rotator cuff tears (IRCTs), thereby reducing the resistance force during shoulder abduction. Our biomechanical study confirmed this hypothesis, as the resistance force during shoulder abduction was lower compared to the IRCT condition. Superior migration of the humeral head is a critical pathology observed in IRCTs. This migration can lead to impingement, higher contact pressure, and an increase in the resistance force during shoulder abduction. Lukas N Muench et al. investigated the impact of an isolated full-thickness supraspinatus (SSP) tear on glenohumeral kinematics and contact mechanics. Their study revealed a significant increase in cumulative deltoid force and glenohumeral contact pressure following an SSP tear, both of which decreased after rotator cuff repair [15].

Various procedures, such as trapezius tendon transfer and superior capsule reconstruction (SCR), have been developed to compress the humeral head and maintain the rotator fulcrum. Gyuna Baek et al. found that irreparable isolated supraspinatus tendon tears (IISTTs) significantly increased superior translation compared to the intact rotator cuff condition. Middle trapezius tendon (MTT) transfer effectively reduced superior translation, peak contact pressure, and mean contact pressure [16]. Lukas N Muench et al. studied SCR in irreparable posterosuperior rotator cuff tears (PSRCT), observing a decrease in glenohumeral abduction angle and an increase in superior humeral head migration and cumulative deltoid force. SCR successfully reduced superior humeral head migration and deltoid forces at specific abduction angles [17].

In our study, soft tissue reconstruction above the humeral head was not performed. Instead, we altered the shape of the greater tuberosity (GT) to create a “ball”-like structure on the proximal humerus. Our hypothesis was that tuberoplasty would decrease impingement between the GT and acromion in IRCTs, subsequently reducing the resistance force during shoulder abduction. Our biomechanical study confirmed a reduction in resistance force after tuberoplasty. In both tuberoplasty testing cycles, the deltoid force was significantly lower than that observed in IRCTs, suggesting a decrease in impingement resistance. The smooth surface and arc structure of the GT contributed to this effect. Tuberoplasty not only reduced total deltoid force but also facilitated a linear shoulder abduction motion. The loading curve exhibited a smoother profile post-tuberoplasty, eliminating the previously observed “bumpier” pattern during IRCTs testing cycles. The disappearance of the bumpier pattern indicated a reduction in impingement resistance between the GT and acromion. The arc structure of the GT and prosthesis played a crucial role in minimizing impingement resistance.

While the present study has provided valuable insights, there are limitations. Future studies using fresh frozen cadavers and enhanced testing equipment are planned to address these limitations. Additionally, further investigation is required to thoroughly assess the suitability of the GT prosthesis for clinical use.

In conclusion, tuberoplasty effectively reduces resistance force during dynamic shoulder abduction in cases of irreparable rotator cuff tears. Notably, prosthesis tuberoplasty does not confer an advantage over burnishing tuberoplasty in terms of resistance force.

## Data Availability

No datasets were generated or analysed during the current study.
